# Curvature of Posterior Pole in Eyes with Retinitis Pigmentosa

**DOI:** 10.3390/jcm13226806

**Published:** 2024-11-12

**Authors:** Masato Kakisu, Gen Miura, Tatsuya Nagai, Ryutaro Akiba, Takayuki Baba

**Affiliations:** Department of Ophthalmology and Visual Science, Chiba University Graduate School of Medicine, Chiba 260-8670, Japan

**Keywords:** retinitis pigmentosa, optical coherence tomography, curvature, choroidal thickness

## Abstract

**Background/Objectives**: This study aimed to determine whether there is a significant change in eyeball curvature in eyes with retinitis pigmentosa (RP). **Methods**: The medical records of 35 eyes of 18 patients with RP and age- and axial-length-matched controls were reviewed. The curvature of the posterior pole was determined by approximating a second-order polynomial equation based on the optical coherence tomography (OCT) images. Associations among eyeball curvature, refractive error, and axial length were investigated. **Results**: The average age of patients with RP was 65.1 ± 13.8 years, and the average axial length of the eye was 23.90 mm. The curvature of the posterior eyeball was steeper in eyes with RP (*p* = 0.020), and the choroid was thinner in eyes with RP (*p* < 0.01). The curvature of eyes with RP significantly correlated with refractive error (*p* = 0.006, *r* = −0.46) and axial length (*p* = 0.004, *r* = −0.48). **Conclusions**: The significant correlation between eyeball curvature and axial length suggests that myopia affects eyeball shape even in eyes with RP. However, the curvature remained steep in the eyes with RP after matching for age and axial length. A thinner choroid was observed in eyes with RP and may play a role in the steeper posterior eyeball.

## 1. Introduction

Retinitis pigmentosa (RP) is an inherited degenerative retinal disease. The prevalence of RP is one in 4000–5000. Patients with RP typically experience night blindness at a younger age, and the symptoms of visual field and vision loss eventually progress to total blindness. To date, the treatment of RP is under investigation, and recently, gene therapies such as gene replacement and editing have become available for very limited types of gene mutations associated with RP [1].

Recent advances in optical coherence tomography (OCT)-based morphological observations of the microstructure of the RP retina have enabled our group to identify changes in the retina even during the early stages of RP [2,3,4]. However, the choroid and sclera in patients with RP have not been investigated in detail. Some groups have reported that the curvature of the eyeball is steeper in RP based on OCT images [5]. A steeper posterior eyeball resembles a staphyloma in high myopia, but the choroid in eyes with RP has not been reported to be as thin as that in eyes with myopia [6]. Whether posterior staphyloma develops progressively in eyes with RP remains controversial. Retinal degeneration may cause choroidal ischemia, leading to posterior staphyloma. In contrast, retinal atrophy is often evident in highly myopic eyes with posterior staphyloma. The close relationship between posterior staphyloma and retinal degeneration makes us aware of a potential strategy to slow retinal degeneration in patients with RP by preventing the development of posterior staphyloma. To proceed with this treatment strategy, further investigation of choroidal and scleral anatomy in eyes with RP is necessary.

This study aimed to perform an age- and axial-length-matched comparison between eyes with RP and controls, and to investigate the significance of posterior staphyloma in eyes with RP. We also studied the relationship between staphylomas and choroidal findings in patients with RP.

## 2. Materials and Methods

This retrospective study included 35 eyes from 18 Japanese patients with RP. Twelve patients were female. Patients were followed up at Chiba University Hospital and data were collected from electronic medical records. Patients with vision-affecting cataracts, postoperative capsule opacity, or eyes with retinal conditions other than RP were excluded. The study protocol was approved by the Institutional Review Board of Chiba University Hospital (No. 5-197), and the procedures conformed to the tenets of the Declaration of Helsinki.

In addition to standard ophthalmological examinations, each patient was examined using spectral-domain OCT (Spectralis OCT; Heidelberg Engineering, Heidelberg, Germany), and the axial length of the eyes was measured using OA-2000 (TOMAY, Aichi, Japan). The mean deviation of retinal sensitivity was measured using a Humphrey visual field analyzer with the 10-2 program (HFA II 750; Carl Zeiss Meditec, Dublin, CA, USA).

**Figure 1:**
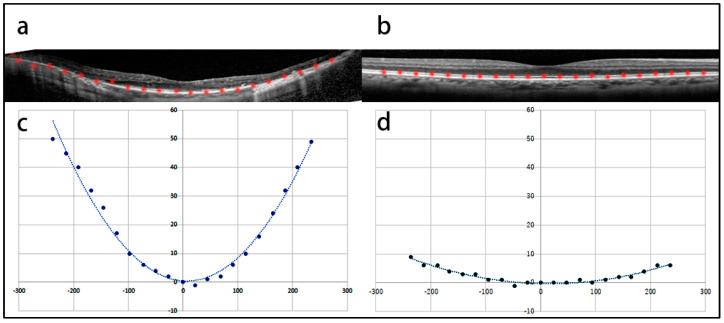
The measurement of the curvature of the eyes with retinitis pigmentosa (RP) and control eyes. (**a**) An optical coherence tomography (OCT) image of an 84−year−old man with RP. The axial length is 27.16 mm. The line of retinal pigmented epithelium (RPE) was marked with red points at an interval of 200 µm. (**b**) An OCT image of the control group. The left eye of an 84−year−old man was examined. The mean axial length of the eye was 27.37 mm. The RPE line is marked in the same manner as in (**a**). (**c**) The curvature of the eye was fitted using NIH Image J software (ver. 1.54g). Coefficient a represents the steepness of the eye and is 9.0 × 10^−4^. (**d**) The curvature of the eye is flat, and coefficient a is 1.0 × 10^−4^.

The posterior eyewall curvature was determined as follows: The horizontal and vertical line scans were recorded through the foveal pit. The images were generated by averaging 100 b-scan images and were exported as JPEG files. The length of each scan was 30°. The highly reflective line of retinal pigment epithelium was plotted at intervals of 200 µm on the horizontal OCT image through the fovea. The plotting was performed in Photoshop CS5 (ver. 12.0, Adobe, San Jose, CA, USA) using the “pen” tool. The interval between plots was measured using a Photoshop grid. The plots were then rotated to the center of the fovea to adjust their alignment by the “rotate image” function of the software. Once the heights of both ends of the plots reached the same level, the image was imported into ImageJ software (ver. 1.54g, NIH, Bethesda, MD, USA), and the x- and y-coordinates were determined. By dragging the cursor on every plot, the x- and y-coordinates were given on the tool window of Image J. Finally, the plots were approximated to the 2nd-order polynomial equation y = ax^2^ + bx + c by using the “curve fitting” function of Image J, and the coefficient a was used to determine the steepness of the eyeball curvature (Figure 1a,c: RP; Figure 1b,d: control). The details of these measurements are reported elsewhere [7].

The central foveal thickness was measured using the caliper function of the Spectralis OCT. The distance between the inner border of the neurosensory retina and inner border of the retinal pigment epithelium was measured manually at the central fovea. Choroidal thickness was also measured using the caliper function of Spectralis OCT. The distance between the outer border of the retinal pigment epithelium and border of the choroid/sclera was recorded as choroidal thickness.

The relationships between eyeball curvature and axial length, refractive error, central foveal thickness, visual acuity, retinal sensitivity, and length of the ellipsoid zone (EZ) were investigated. We also compared RP cases with age- and axial-length-matched controls. We adjusted for these two factors because the prevalence of posterior staphyloma increases with age [8] and axial length [9]. There were 12 eyes in each group and the same measurements were performed. We matched two factors, the patient’s age and axial length, and this two-factor matching was challenging, especially in young patients. We reviewed and matched 12 cataract patients who underwent surgery in the last two years. Differences in eyeball curvature and choroidal thickness were assessed between the two groups.

The Spearman test was used to determine the relationship between curvature, axial length, and refractive error. The Mann–Whitney U test was used to determine the difference between the curvature of the eye and choroidal thickness in each group. Statistical significance was set at *p* < 0.05. All statistical analyses were performed using SPSS ver. 20 (IBM Corp., Armonk, NY, USA). The propensity score was calculated for the RP group and controlled using EZR in R commander ver. 1.63 (Saitama Medical Center, Jichi Medical University, Saitama, Japan).

## 3. Results

The average age of 18 RP subjects was 60.9 ± 18.3 years (27–84). The average axial length, visual acuity, spherical equivalent refractive error, eyeball curvature (coefficient a), central foveal thickness, choroidal thickness, and foveal EZ length are shown in Table 1.

**Figure 2:**
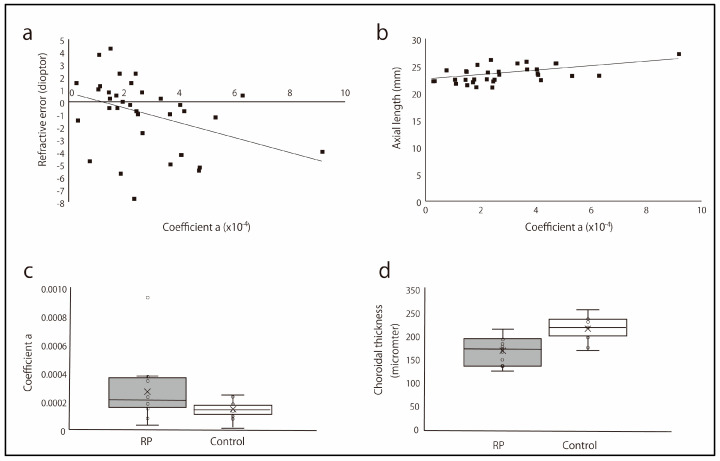
Curvature of eyeball and its relationship with refractive error and axial length. (**a**) The relationship between the curvature of the eyeball and refractive error. There was a significant negative correlation between the curvature of the eyeball and refractive error (*p* = 0.006, *r* = −0.456). (**b**) The relationship between the curvature of the eyeball and axial length. There was a significant relationship between eyeball curvature and axial length (*p* = 0.004, *r* = 0.477). (**c**) The curvature of the eyeball in the eyes with retinitis pigmentosa (RP) and controls. The curvature of the eyeball was significantly steeper in the eyes with RP than in the control eyes (*p* = 0.020). ×: median. (**d**) The choroidal thickness of the eyeball in the eyes with RP and controls. The choroids of the eyes were significantly thinner than those of the control eyes (*p* < 0.01). ×: median.

There was a significant negative correlation between refractive error and curvature of the eyeball (*p* = 0.006, *r* = −0.456; Figure 2a). There was also a significant positive correlation between the axial length and curvature (*p* = 0.004, *r* = 0.477, Figure 2b). In contrast, there was no significant correlation between the curvature and central foveal thickness (*p* = 0.907, *r* = 0.021), length of the macular ellipsoid zone (*p* = 0.496, r = −0.119), visual acuity (*p* = 0.107, *r* = −0.282), and mean deviation of retinal sensitivity (*p* = 0.904, *r* = −0.022).

The average age in the 12 RP and 12 age- and axial-length-matched control eyes was 65.1 ± 12.8 (95% CI: 56.5–73.7), for the range 44–84 years, and 66.4 ± 12.7 (95% CI: 58.3–74.5), for the range 45–84 years (*p* = 1). The average axial length in RP and control was 23.90 ± 2.17 (95% CI: 22.4–25.4), for the range 21.0–27.2 mm, and 23.97 ± 1.99 (95% CI: 22.7–25.2), for the range 21.6–27.4 mm (*p* = 0.977). The standardized differences in age and axial length were 0.116 and 0.044, respectively, after propensity score matching using a caliper of 0.2. When we compare the curvature and choroidal thickness between the two groups, the curvature of the eyeball was significantly steeper in the RP eyes than that in controls (2.90 ± 2.31 × 10^−4^ vs. 1.36 ± 0.63 × 10^−4^, *p* = 0.020, Figure 2c). The range of curvature was between 0.33 and 9.2 × 10^−4^ (95% CI: 1.35–4.46 × 10^−4^) in the RP eyes and between 0.10 and 2.39 × 10^−4^ (95% CI: 0.96–1.76 × 10^−4^) in controls. The choroid of RP eyes was significantly thinner than that in the control eyes (195 ± 33 vs. 249 ± 28 µm, *p* < 0.01, Figure 2d). The range of choroidal thickness was between 146 and 248 µm (95% CI: 173–217) in the RP eyes and was between 198 and 295 µm (95% CI: 231–267) in controls.

## 4. Discussion

In this study, we observed a steep curvature of the posterior eyeball in eyes with RP, based on OCT images. After matching for age and axial length, the curvature remained significantly steeper and the choroidal thickness was thinner in eyes with RP than in control eyes.

In highly myopic eyes, the axial length is elongated and the choroid becomes thinner following the elongated axial length [10]. This change is more evident in the posterior part of the eyeball, and the posterior staphyloma is known to outpouch the posterior fundus. The choroid is remarkably thin in posterior staphylomas, and impaired blood circulation in the choriocapillaris can lead to degenerative changes in the retinal pigment epithelium and photoreceptors [11]. Finally, severe retinal atrophy develops in staphylomas, resulting in poor visual function [12]. In this study, we observed a steeper eyeball curvature in the RP group. The border of the posterior staphyloma is unclear; however, diffuse changes are observed in the posterior eyeballs. Choroidal thickness was also lower in eyes with RP. Therefore, decreased blood flow can cause ischemia in the sclera and accelerate scleral remodeling [13]. The mechanism of scleral thinning after ischemia has not been fully elucidated; however, the recruitment of MMP (Matrix metalloproteinase)-2-driven fibroblasts contributes to the irregular production of collagen-1, resulting in thinner collagen fibers [14]. This remodeling of scleral collagen may cause the expansion of the posterior eyeball in eyes with RP.

Scleral expansion and choroidal thinning are potential targets for RP treatment. Retinal pigment epithelium atrophy develops in the eyes of patients with posterior staphyloma or high myopia. Although these changes progress gradually, retinal damage can be caused by posterior staphyloma and choroidal thinning in eyes with RP. Low-emission red light has also been reported to exert a protective effect against myopia progression by increasing choroidal blood flow [15]. Such treatment may prevent thinning of the choroid, scleral expansion, and slow retinal degeneration in patients with RP.

The change in retinal thickness was less than that in the sclera of the eyes with high myopia [16]. Similarly, retinal thickness was comparable between the RP and control eyes in our study. In eyes with RP, the central macula is preserved until the end of the disease. This finding suggests that retinal damage around the fovea is not directly caused by choroidal dysfunction. Further investigations are necessary to determine the relationship between choroidal thickness and retinal function in patients with RP.

Posterior staphylomas have also been reported [17]. We determined the presence of a posterior staphyloma by fundus observation using conventional ophthalmoscopy and swept-source OCT. Posterior staphyloma was diagnosed based on subjective observation. We used the curvature measured using OCT to eliminate the subjective view and for a more precise and objective assessment of the shape of the posterior eyeball in eyes with RP. As the calculation of the curvature to evaluate the severity of posterior staphyloma is quantifiable, further analysis, such as the relationship between posterior staphyloma and retinal function, is required. Retinal function in the RP eyes includes not only visual acuity but also retinal sensitivity measured by microperimetry, retinal response by electroretinogram, metamorphopsia, and contrast visual acuity. If the strategy to reduce the posterior staphyloma in the RP eyes becomes implementable, those potential evaluations are of substantial value to measure the effectiveness of the treatments.

A major limitation of our study was the small number of patients. Cases of RP are relatively rare, and recruiting many cases is difficult, although our RP clinic has a considerable number of patients. Matching the age and axial length is challenging, particularly in young patients. Additionally, functional assessments of patients with RP have not been thoroughly performed. Visual acuity was not associated with eyeball curvature; however, other measurements, such as retinal sensitivity with microperimetry, may be useful for evaluating the relationship between curvature and retinal function in a wider field.

## 5. Conclusions

The significant correlation between eyeball curvature and axial length suggests that myopia affects eyeball shape even in eyes with RP. However, the curvature remained steep in the eyes with RP after matching for age and axial length. Therefore, underlying mechanisms may affect the shape of the posterior eyeball in patients with RP. A thinner choroid was observed in eyes with RP and may play a role in the steeper posterior eyeball. It is important to investigate the relationship between retinal function determined by multimodal imaging and the curvature of the eyeball in RP to further understand disease pathology.

## Figures and Tables

**Table 1:** Patients demographics.

	Mean ± SD	Range	95% CI
Axial length (mm)	22.0 ± 6.01	21.0, 27.2	19.9, 24.1
Visual acuity (logMAR)	0.20 ± 0.30	−0.08, 1.39	0.10, 0.31
SER (D)	−1.03 ± 2.9	−7.75, 4.25	−2.01, −0.05
Curvature of eyeball	2.75 ± 1.82 × 10^−4^	0.28, 9.18 × 10^−4^	2.1, 3.3 × 10^−4^
CFT (micrometer)	186 ± 51	30, 278	169, 203
CT (micrometer)	179 ± 50	89, 289	161, 197
EZ (micrometer)	3900 ± 2689	781, 8505	2896, 4904

SD: standard deviation; CI: confidence’ interval; SER: spherical equivalent refractive error; CFT: central foveal thickness; CT: choroidal thickness; EZ: ellipsoid zone.

## Data Availability

The raw data supporting the conclusions of this article will be made available by the authors on request.
